# Blockade of telomerase reverse transcriptase enhances chemosensitivity in head and neck cancers through inhibition of AKT/ ERK signaling pathways

**DOI:** 10.18632/oncotarget.5468

**Published:** 2015-10-16

**Authors:** Tengda Zhao, Fengchun Hu, Xingguang Liu, Qian Tao

**Affiliations:** ^1^ Department of Oral and Maxillofacial Surgery, Guanghua School of Stomatology, Hospital of Stomatology, Sun Yat-sen University, Guangdong Provincial Key Laboratory of Stomatology, Guangzhou, Guangdong, China; ^2^ Department of Oral and Maxillofacial Surgery, Provincial Hospital Affiliated to Shandong University, Jinan, Shandong, China; ^3^ Department of Stomatology, The First Affiliated Hospital of Guangzhou University of Chinese Medicine, Guangzhou, Guangdong, China

**Keywords:** TERT, HNSCC, AKT, ERK, chemosensitivity

## Abstract

Head and Neck squamous cell carcinomas (HNSCC), characterized by the high frequency of local recurrence and distant metastases, is mostly related to highly malignant and resistant to apoptosis, resulting in significant insensitivity to chemotherapy. Telomerase reverse transcriptase (TERT), as the catalytic subunit of telomerase, was implicated in the telomerase-mediated cellular transformation, proliferation, stemness and cell survival. Moreover, overexpression of human TERT (hTERT) is reported to be correlated with advanced invasive stage of the tumor progression and poor prognosis. Here, we show that hTERT potentially mediated the apoptotic resistance and blockade of telomerase reverse transcriptase could enhance chemosensitivity in head and neck cancers. Mechanistically, hTERT interacts with the phosphorylation of AKT and ERK to suppress the expression of p53, ultimately, leading to modulation of the cellular sensitivity to chemotherapy. Thus, these findings suggest that hTERT targeting could be an attractive approach in combination with conventional chemotherapies for patients suffering from chemoinsensitivity or refractory HNSCC.

## INTRODUCTION

Head and Neck squamous cell carcinomas (HNSCC), which arise in the oral cavity, larynx and pharynx, ranks as the sixth most prevalent cancer with approximately 500,000 new cases per year worldwide [1]. Despite advances in treatment, HNSCC patients often present with advanced-stage tumors and the five-year survival rate is less than 50% [2, 3], this poor prognosis is mostly related to high malignancy and resistance to apoptosis, resulting in significant insensitivity to chemotherapy [4]. Therefore, investigation of the potential key mediators that regulate chemosensitivity may help to further uncover the biological basis of HNSCC and improve clinical therapy.

Telomerase is an enzyme best known for its role in telomere maintenance [5]. Telomerase reverse transcriptase (TERT), as the catalytic subunit of telomerase, has the capacity to limit the activity of telomerase to avoid the erosion of telomere. Several studies have demonstrated that human TERT (hTERT) was implicated in the telomerase-mediated cellular transformation [6], proliferation [7], stemness [8] and cell survival [9]. It is well established that introduction of hTERT into fibroblasts and some epithelial cells confers cellular immortalization [10–12], and hTERT reactivation is a critical step for transformed cells during malignant transformation. Moreover, overexpression of hTERT is reported to be correlated with advanced invasive stage of the tumor progression and poor prognosis [13–16].

Cancer cells tend to survive despite violating rules of normal cellular behavior that ordinarily provoke apoptosis. Accumulating studies reported that hTERT positively impacts mitochondrial function to modulate cell survival in response to apoptotic stimuli. Furthermore, a recent study has showed that hTERT overexpression alleviated intracellular reactive oxygen species (ROS) production and inhibited ROS-mediated apoptosis [17]. These properties suggest that hTERT might influence the sensitivity of chemotherapy in cancer cells, although the precisely molecular mechanisms are still largely unknown.

Recent studies have significantly increased our understanding that the p53 family and various pathways played a major role in DNA-damage responses [18]. Another report also revealed that AKT mediated cisplatin resistance by modulation of p53 action on caspase-dependent mitochondrial death pathway in ovarian cancer [19]. However, it remains to be established how these various pathways are differentially activated and how they might be interconnected during chemotherapy.

In the present study, we demonstrated that hTERT potentially mediated the apoptotic resistance and blockade of telomerase reverse transcriptase could enhance chemosensitivity in head and neck cancers. Most importantly, we found the mechanism that hTERT interacts with the phosphorylation of AKT and ERK to suppress the expression of p53, ultimately, leading to modulation of the cellular sensitivity to chemotherapy. Taken together, our results suggest that the AKT/ERK-p53-bcl-2 axis might constitute a signaling cascade that mediates the antitumor treatment in HNSCC cells, and hTERT targeting could be an attractive approach in combination with conventional chemotherapies for patients suffering from chemo-insensitivity or refractory HNSCC.

## RESULTS

### hTERT is activated to modulate cells survival in response to dead signal

**Figure 1:**
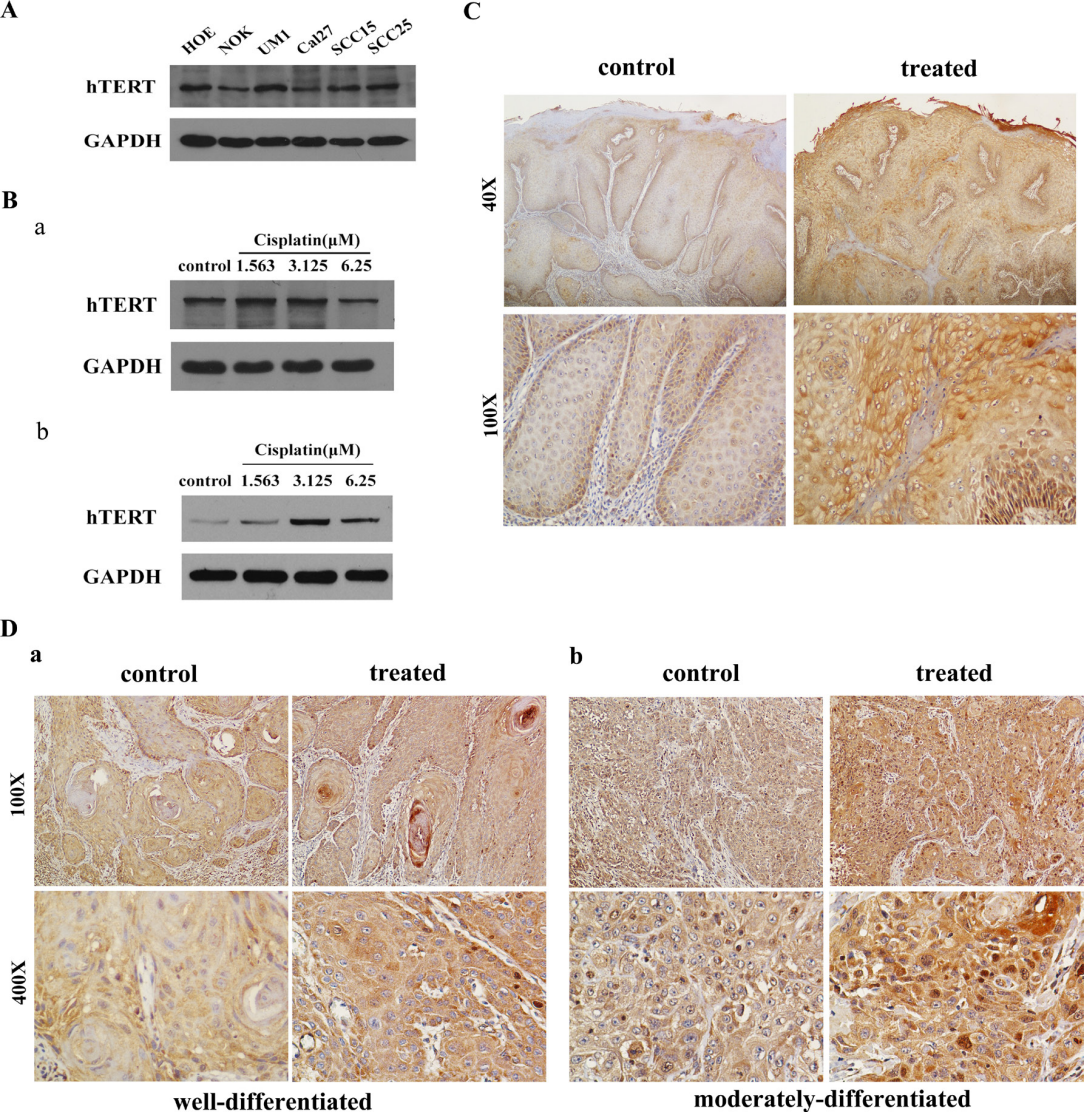
hTERT is activated in cells undergoing apoptotic stimuli and primary human HNSCC tissues treated with induction chemotherapy **A.** Western blot analysis of hTERT expression in primary cell line HOE, immortalized normal cell line NOK and various HNSCC cell lines using anti-hTERT antibodies. Anti-GAPDH served as loading control. **B.** Expression of hTERT in HNSCC cell lines Cal27 a. and SCC25 cells b. were analyzed in the presence of a range of different concentrations of chemotherapy drugs by Western blot analysis. Anti-GAPDH was used as loading control. **C, D.** Immunohistochemical staining of hTERT expression in oral epithelium (C) and well to moderately-differentiated squamous tumor nests (D) of HNSCC tissues treated with or without chemotherapy.

High levels of TERT expression and telomerase activation have been reported in many cancer cells [20, 21]. Our previous study also demonstrated that hTERT is overexpressed in oral epithelial dysplasia and oral squamous cell carcinoma (OSCC) tissues and correlates with clinical aggressiveness of OSCC patients. Further detection by Western blot was performed to find that hTERT expression was significantly upregulated in frequently utilized HNSCC cells compared with the immortalized normal keratinocyte cell line (NOK) (Figure 1A), suggesting hTERT might act as a strong promoter in malignant development of HNSCC.

It is known that hTERT expression and telomerase activity are often very low or undetectable in somatic cells *in vivo*. However, our results showed that hTERT was expressed at a high level in primary human oral epithelial cells (HOE) *in vitro* (Figure 1A), which enlightened us that hTERT might confer protective activity to resist deadly damage. We further speculated that hTERT might modulate the cancer cell survival upon chemotherapy, then the changes of hTERT were tested in two HNSCC cells treated with cisplatin at different concentrations, as shown in Figure 1B, the expression of hTERT increased in a dose-dependent manner at certain range of concentration, which suggested that hTERT might be an essential determinant for tumor cells escape from death.

Next, to observe the effect of hTERT on cellular self-protection to chemotherapeutic drugs clinically, immunohistochemical staining was performed to detect the expression of hTERT in HNSCC tissues treated with or without induction chemotherapy. Compared to the patients without treatment, we found that the expression of hTERT significantly increased in patients who had received chemotherapy. For HNSCC samples without treatment, moderate to strong cytoplasmic and nuclear hTERT staining was exhibited in nearly all epithelial cells, while more marked cytoplasmic and nuclear hTERT staining was observed in HNSCC treated with drugs. In addition, cells from central parts of the nests from well to moderately-differentiated squamous tumors showed the same uptrend between them. Representative microphotographs of hTERT staining for HNSCC with or without induction chemotherapy are shown in Figure 1C and 1D. The mean cytoplasmic hTERT LSs in epitheliums and central nests separately increased significantly from HNSCC control group (187.65% ± 10.698%, 163.47% ± 11.193%, 191.60% ± 30.835%) to HNSCC samples with chemotherapy (267.23% ± 18.910%, 205.64% ± 37.272%, 276.00% ± 20.236%) (Table 1). The mean nuclear hTERT LSs also increased from (200.65% ± 32.843%, 154.00% ± 13.130%, 202.60% ± 40.072%) to (258.00% ± 10.882%, 204.00% ± 38.508%, 263.4% ± 15.060%), there was a significant difference in the mean cytoplasmic and nuclear hTERT LSs before and after chemotherapy (*P* < 0.05). Collectively, these results suggest that hTERT plays an important role in cell survival when receiving death stimuli.

**Table 1:** The mean hTERT LS in HNSCC samples treated with or without chemotherapy

Groups	Mean cytoplasmic hTERT LS ± SD (%)	*P*-value	Mean nuclear hTERT LS ± SD (%)	*P*-value
**Epithelium**				
Control	187.65 ± 10.698		200.65 ± 32.843	
Treated	267.23 ± 18.910	0.000	258.00 ± 10.882	0.000
**Nest**				
WD HNSCC				
Control	163.47 ± 11.193		154.00 ± 13.130	
Treated	205.64 ± 37.272	0.001	204.00 ± 38.508	0.000
MD HNSCC				
Control	191.60 ± 30.835		202.60 ± 40.072	
Treated	276.00 ± 20.236	0.001	263.4 ± 15.060	0.024

Comparison between groups was performed by Student's *t*-test. A significant difference in the mean cytoplasmic and nuclearhTERT LS was found in epithelium and nest between OSCC control and chemotherapy treated groups (*P* < 0.05). Labeling scores (LS); Head and neck squamous cell carcinoma (HNSCC); WD = well-differentiated, MD = moderately-differentiated.

### hTERT potentially activates p53-mitochondrial pathway to contribute to death escape

**Figure 2:**
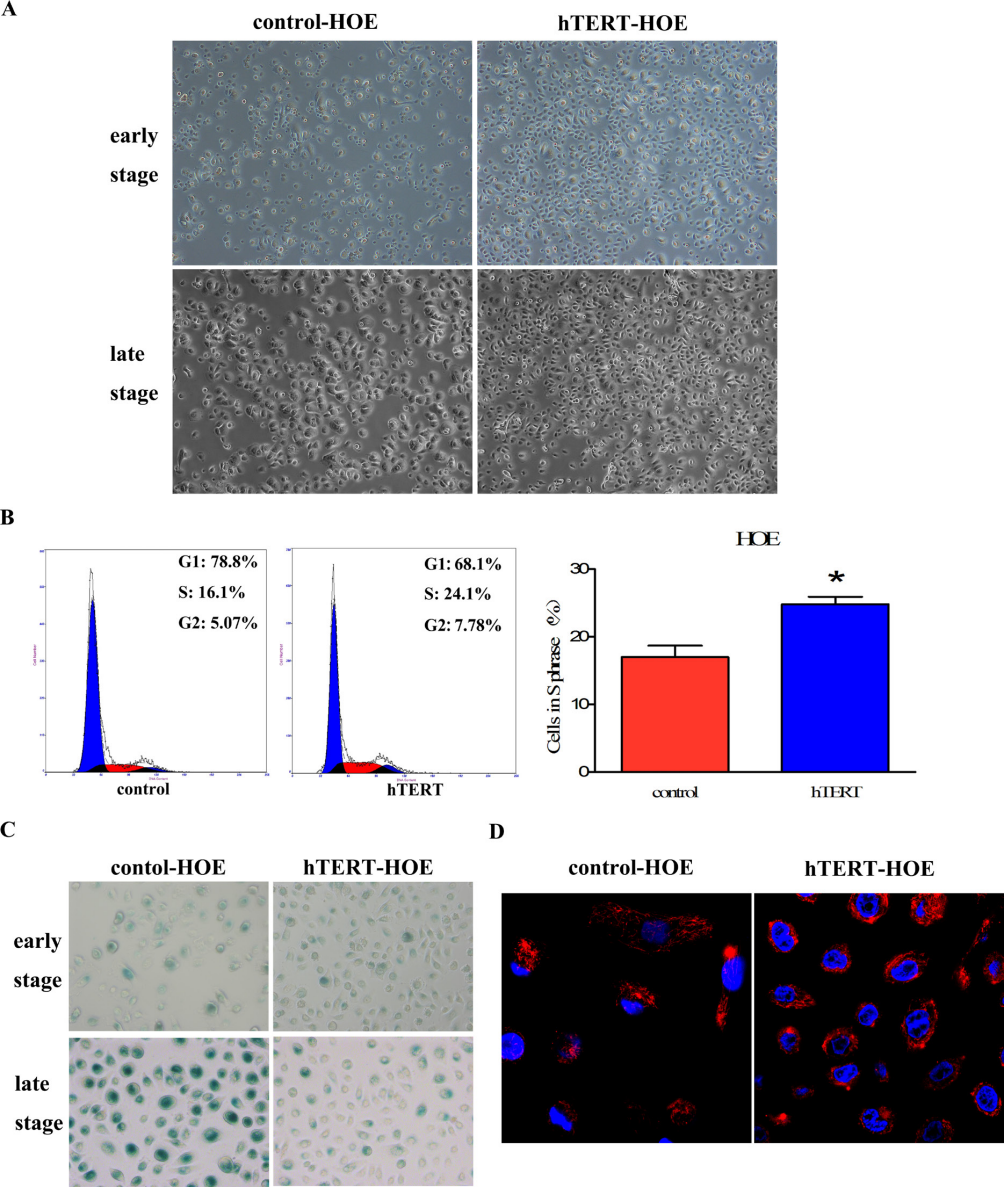
hTERT promotes cells survival and improves mitochondrial function in primary HOE cells **A.** The cellular morphology of HOE cells expressing either the control vector pCMV or pCMV/hTERT at the early and late passages (passage 15 and 25, respectively) was shown by phase contrast micrography, flattened morphology are indicative of senescence. Scale bar: 200 μm. **B.** Effect of hTERT on the viability status of HOE cells expressing either the control vector pCMV or pCMV/hTERT was measured by cell-cycle analysis. *P < 0.05 as compared with the control vector cells. **C.** The SA–Δ galactosidase activity was tested in the early (passage 17) and later (passage 32) stages to determine the senescent conditions of pCMV vector or pCMV/hTERT HOE cells. Blue-stained cells were identified as senescent cells. Scale bar: 50 μm. **D.** Identification was examined by confocal microscopy, red-positive staining of mitochondria in the HOE cytoplasm, while the blue signal signifies nuclear DNA staining with DAPI. Scale bar: 10 μm.

To better clarify the biological function of hTERT in response to cell death, we established stably hTERT-expressing cells using the HOE cells to exclude the involvement of other mutant genes. As shown in Figure 2A, we observed that the adherent cells showed a typical small cobblestone-like epithelial morphology in an early phase. However, with the later process of serial passages, HOE cells infected with the control lentivirus underwent senescence and represented flatten morphology, while hTERT-overexpressing cells remained remarkably unchanged. Cell-cycle analysis was performed to determine these cells’ status. Indeed, the percentage of control vector cells in S phase (16.989% ± 1.689%) was lower compared to hTERT-overexpressing cells (24.776% ± 1.142%)(Figure 2B). This was further supported by detection of the SA–Δ galactosidase activity (Figure 2C). In addition, mitochondrion, the key mediator of apoptosis, is activated upon dead signal to resist cell death, we then evaluated the effect of hTERT-overexpressing on mitochondrial activation using MitoTracker^®^ Red dye. As shown in Figure 2D, active labeled mitochondria were frequently observed in HOE cells that overexpressed hTERT, which suggested that hTERT could affect mitochondrial pathway to protect cells from senescence.

Increasing evidence implicated that p53 is a central factor in the cellular response to damaging stimuli to regulate cell apoptosis through mitochondria [22]. We previously determined the expression of p53 by western blot analysis and found it significantly reduced in hTERT-overexpressing HOE cells [23], these results indicate that hTERT could activate p53-mitochondrial pathway to promote mitochondrial function, which helps to prevent cell death. Collectively, according to the previous two parts of results, we deduced that hTERT might play a crucial role in modulating the cellular sensitivity to chemotherapy.

### Ectopic expression of hTERT enhances chemoresistance by activating AKT and ERK signaling pathways in cancer cells

**Figure 3:**
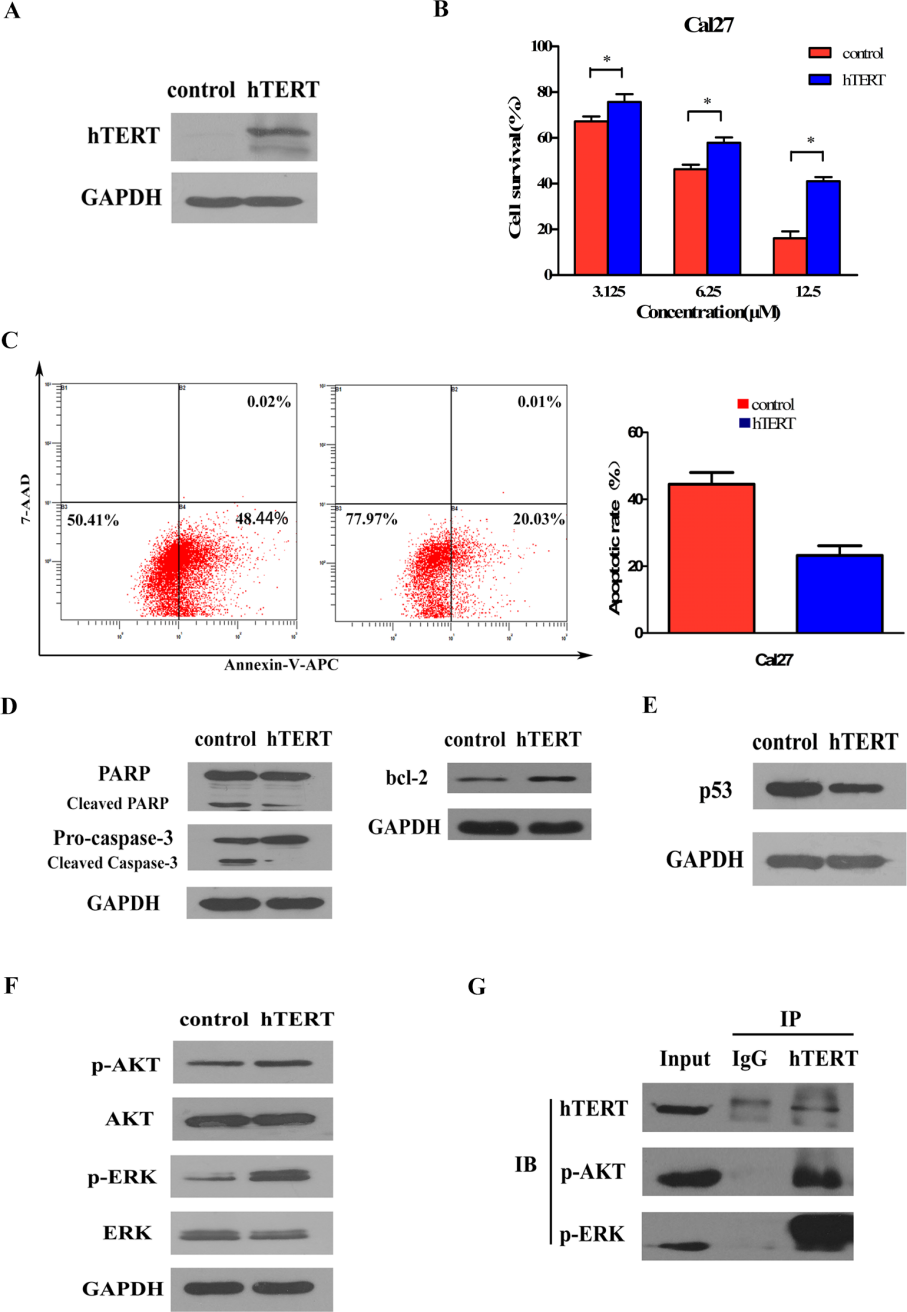
Ectopic expression of hTERT suppresses drug sensitivity by activating AKT and ERK signaling pathways in cancer cells **A.** Ectopic expression of hTERT in Cal27 cell line. Total cell lysates of control (pCMV) and hTERT-overexpressing (pCMV/hTERT) cells were analyzed by Western blot analysis using anti-hTERT antibodies. Anti-GAPDH was used as loading control. **B.** hTERT expression prevents cisplatin-induced cell death. 48 h after treatment with cisplatin, cell viability was assessed by MTT method. Bars represent the average percentages of survival cells compared with the untreated control cells; *P < 0.05. **C.** uantification of Annexin V-APC/7-AAD cells after the indicated cells were treatment with cisplatin (6.25 μM) for 48 h. Each bar represents the mean ± SD of three independent experiments; *P < 0.05. **D.** hTERT expression inhibits cisplatin-induced proteolysis of pro-caspase-3 and PARP and induced bcl-2 expression. The Cal27 cells treated with cisplatin (6.25 μM) for 48 h and the total cell lysates were analyzed for proteolysis cleavage of pro-caspase-3, PARP (left) and expression of bcl-2 (right) by Western blot analysis. Anti-GAPDH was used as loading control. **E.** Repression of p53 pathway in hTERT-overexpressing Cal27 cells and control vector Cal27 cells without cisplatin treatment were analyzed by Western blot analysis using antibodies against p53. Anti-GAPDH was used as loading control. **F.** Ectopic expression of hTERT activates the AKT/ERK pathway. The total cell lysates were analyzed for phosphorylated AKT, total AKT, phosphorylated ERK and total ERK levels by Western blot analysis. Anti-GAPDH was used as loading control. **G.** hTERT interacts with p-AKT and p-ERK to regulate the expression of p53. Endogenous phosphorylationof AKT and ERK bound to hTERT immunoprecipitates were analyzed by immunoprecipitation (IP) assays. hTERT immunoprecipitation was done from whole cell lysates of Cal27 cells. IgG served as control.

Escape from apoptosis is an almost systematic hallmark of cancer cells that contributes to tumor progression and drug insensitivity [24]. The discovery of hTERT-mediated self-protection enlightened us that hTERT overexpression might endow cancer cells with a survival advantage by blunting apoptotic signals and consequently affected the sensitivity of cancer cells to chemotherapy. To characterize the impact of hTERT on anti-apoptosis effect, we chose cisplatin, one of the most effective anticancer drugs used in the treatment of HNSCC, and cisplatin-sensitive HNSCC cell line Cal27 which had a low background of hTERT. Next, ectopic hTERT was stably expressed in Cal27 cells (Figure 3A) and the impact of hTERT upregulation on cytotoxic reagent-induced cell apoptosis was evaluated by exposing these cells to cisplatin. As shown in Figure 3B, Cal27 cells expressing ectopic hTERT showed lower degree of cytotoxicity response to various concentration of cisplatin compared with the vector control cells. Annexin-V-APC/7-AAD apoptosis detection was performed by flow cytometry to detect the apoptotic difference. As shown in Figure 3C, the proportion of vector control cells undergoing early and late apoptosis were significantly higher than that of the apoptotic hTERT-expressing cells (44.51% ± 3.510% vs 23.21% ± 2.895%) after cisplatin (6.25 μM) treatment. Expression of bcl-2 and cleavage of caspase-3 and PARP protein critical for cell apoptosis were detected to further demonstrate our observation. As shown in Figure 3D, Cal27 cells expressing ectopic hTERT showed lower levels of cleavages of both pro-caspase-3 and PARP and increased bcl-2 expression.

As we have found that hTERT downregulated p53 in HOE cells, we next examined whether hTERT has the same effect in tumor cells. As shown in Figure 3E, ectopic expression of hTERT also markedly reduced p53 expression. Since the regulation effect of hTERT on p53 and meanwhile they mainly co-localized in the nucleoplasm, we thus asked whether existed the physical interaction between hTERT and p53. However, we failed to find the endogenous interaction by Co-immunoprecipitation (Co-IP) analysis, which suggested that hTERT indirectly regulated p53 through other signaling pathways. We next analyzed the activity of phosphorylated AKT and ERK, upstream molecules of p53 [25–27]. Strikingly, as shown in Figure 3F, overexpression of hTERT clearly increased the phosphorylation of AKT and ERK in Cal27 cells as well as in HOE cells (Supplementary Figure S1). Moreover, endogenous p-AKT and p-ERK bound to endogenous hTERT could be detected in hTERT precipitates by co-IP assays (Figure 3G), suggesting that activation of AKT and ERK was involved in hTERT-induced downregulation of p53 upon death stimuli.

### hTERT suppresses the apoptotic sensitivity of cancer cells *in vivo*

**Figure 4:**
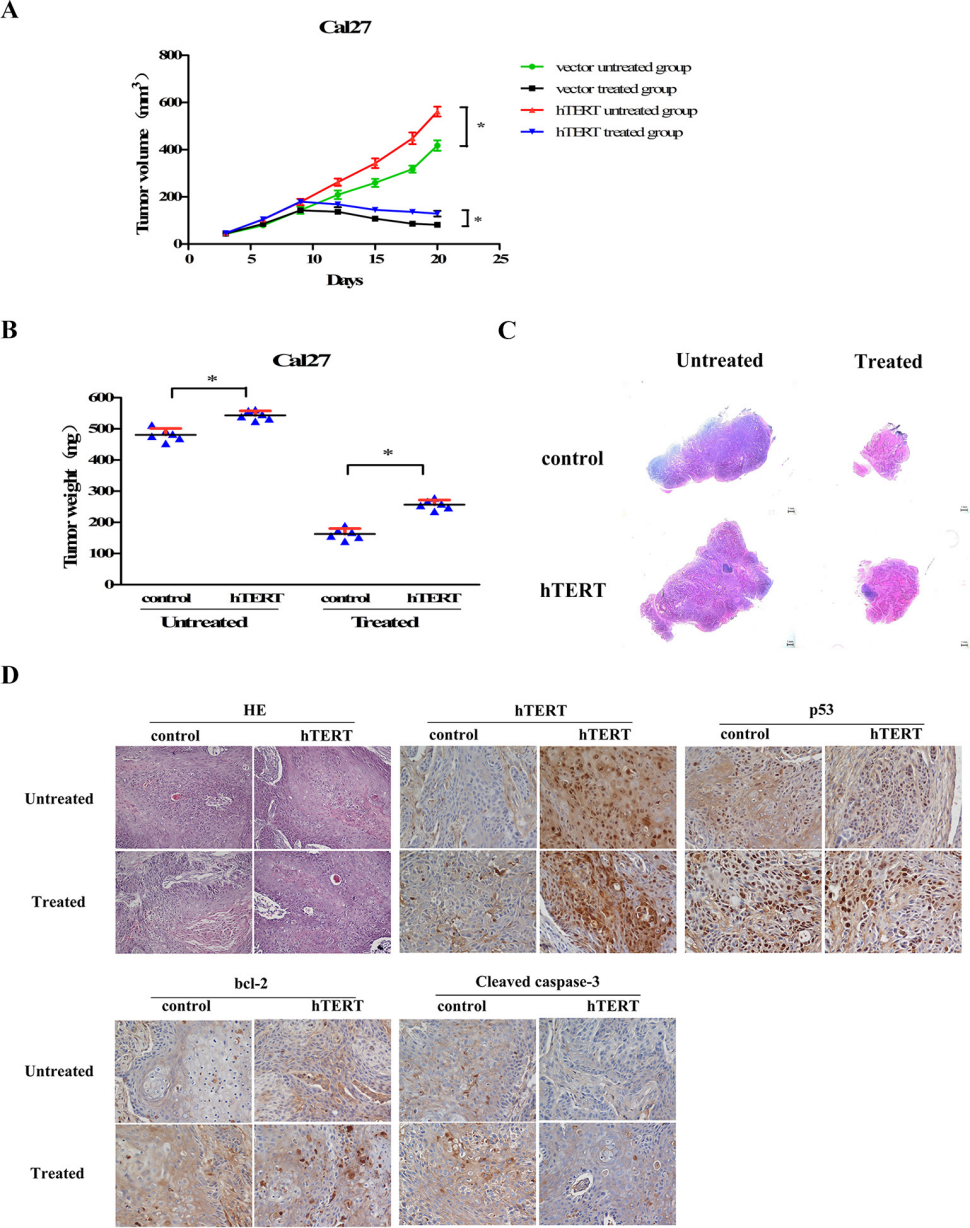
hTERT suppresses the cisplatin sensitivity of cancer cells *in vivo* A total of 1 × 10^7^ control vector or hTERT-overexpressing Cal27 cells were injected subcutaneously into the right flank of each mouse, two different kinds of tumor bearing mice were randomized respectively into two groups after 10 days, and treated by intraperitoneal injection (ip) with cisplatin (2 mg/kg) or control diluent every day. **A.** Xenograft tumor volumes from the mice treated with or without cisplatin were measured on the indicated days; *P < 0.05. **B.** Mean tumor weights for each group; *P < 0.05. **C.** Representative low magnification histological sections from each group. Scale bars: 1 mm. **D.** Representative histological sections of tumor xenografs staining by H & E (Original magnification, 200X) and immunohistochemistry of hTERT, p53, bcl-2, cleaved-caspase-3 each group (Original magnification, 400X).

To further confirm whether ectopic expression of hTERT suppressed the sensitivity to chemotherapy *in vivo*, we next examined effects of hTERT on Cal27 cell line using the nude mouse xenograft model. As shown in Figure 4A–4C, the tumors derived from hTERT-overexpressing Cal27 cells grew more rapidly and were larger in size in contrast to the tumors derived from control vector cells. Moreover, the tumors established using hTERT-overexpressing Cal27 cells were more resistant to apoptosis and formed larger tumors than control cells in mice treated with cisplatin, H & E staining also confirmed this result (Figure 4D). Furthermore, immunohistochemistry (IHC) of tumor biopsies showed increased hTERT expression in tumor xenografts, resulting in decreased level of p53 and upregulation of bcl-2 expression, subsequently suppression of cleavages of pro-caspase-3. Importantly, this trend was more remarkable in hTERT-overexpressing Cal27 cells with cisplatin treatment as compared with control vector cells (Figure 4D). Collectively, these results indicate the critical role of hTERT in conferring resistance ability to chemotherapy *in vivo*.

### Depletion of hTERT sensitizes cancer cells to chemotherapy and inhibited AKT/ ERK signaling pathways

**Figure 5:**
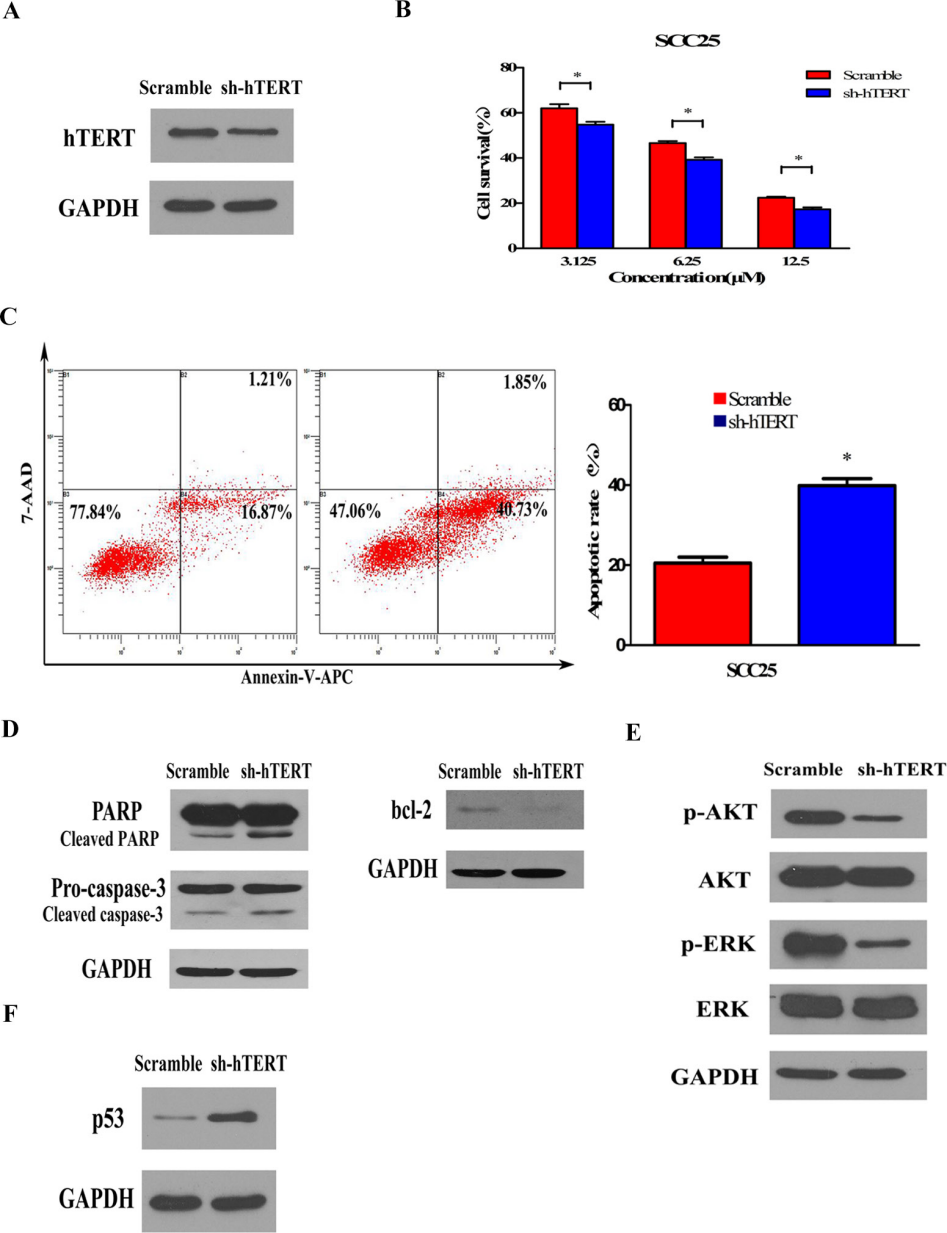
Silencing endogenous hTERT expression sensitizes cancer cells to chemotherapy and inhibited AKT/ ERK signaling pathways **A.** Knockdown of hTERT in shRNA-stably-transduced SCC25 cell line. Total cell lysates of control scramble vector and hTERT-RNAi cells were analyzed by Western blot analysis using anti-hTERT antibodies. Anti-GAPDH was used as loading control. **B.** Knockdown of hTERT enhances the sensitivity to cisplatin-induced cell death. 72 h after treatment with cisplatin (6.25 μM), cell viability was assessed by MTT method. Bars represent the average percentages of survival cells compared with the untreated control cells; *P < 0.05. **C.** Quantification of Annexin V-APC/7-AAD cells after the indicated cells were treatment with cisplatin (6.25 μM) for 72 h. Each bar represents the mean ± SD of three independent experiments; *P < 0.05. **D.** Depletion of hTERT promotes cisplatin-induced proteolysis of pro-caspase-3 and PARP and decreased bcl-2 expression. The Cal27 cells treated with cisplatin (6.25 μM) for 72 h and the total cell lysates were analyzed for proteolysis cleavage of pro-caspase-3, PARP (left) and expression of bcl-2 (right) by Western blot analysis. Anti-GAPDH was used as loading control. **E.** Depletion of hTERT inhibits the AKT/ERK pathway. The total cell lysates were analyzed for phosphorylated AKT, total AKT, phosphorylated ERK and total ERK levels by Western blot analysis. Anti-GAPDH was used as loading control. **F.** Activation of p53 pathway in hTERT-silencing Cal27 cells and control scramble vector Cal27 cells without cisplatin treatment were analyzed by Western blot analysis using antibodies against p53. Anti-GAPDH was used as loading control.

Since the remarkable influence of hTERT to chemosensitivity, we thus considered whether inhibition of hTERT could enhance the chemosensitivity in hTERT-overexpressing cancer cells. We firstly knocked down endogenous hTERT in HNSCC cell line SCC25 that normally expressed high level of hTERT and was insensitive to treatment with cisplatin, then examined the sensitivity of the modified cells to apoptosis. As shown in Figure 5A, one shRNA targeting hTERT had high efficiency to specifically knock down endogenous hTERT protein in SCC25 cells. When the modified cells were treated with 6.25 μM cisplatin, enhanced sensitivity to these apoptotic inducers were found in cells depleted in hTERT compared with vector control cells (Figure 5B). The apoptotic nature of induced cell death was also confirmed by Annexin-V-APC/7-AAD apoptosis detection on hTERT-depleted cells (39.89% ± 2.935%) and control cells (20.54% ± 2.571%) treated with 6.25 μM cisplatin (Figure 5C). In addition, inhibition of hTERT markedly induced cleavage of PARP and pro-caspase-3 and attenuated expression of bcl-2 (Figure 5D). Furthermore, silencing endogenous hTERT decreased the level of phosphorylation of AKT and ERK (Figure 5E), while up-regulated the expression of p53 (Figure 5F), suggesting that silencing endogenous hTERT induced upregulation of p53 through inhibition of AKT and ERK signaling pathways to enhance sensitivity to chemotherapy. Taken together, these results suggest that cellular depletion of hTERT improves the chemotherapy and could be a target in the treatment of chemoinsensitive cancers.

## DISCUSSION

HNSCC, characterized by the high frequency of local recurrence and distant metastases, is mostly related to highly malignant and resistance to apoptosis, resulting in significant insensitivity to chemotherapy. Therefore, improving the sensitivity of the advanced-stage patients to chemotherapy drugs is an important strategy in HNSCC treatment.

The properties of hTERT were correlated with advanced invasive stage of the tumor progression and poor prognosis [15, 16, 28]. Ectopic expression of hTERT has been reported in various types of tumors including lung cancer [29], gastric cancer [30], hepatocellular cancer [31] and breast cancer [32]. In HNSCC, our data of ectopic hTERT expression in clinical samples and cancer cells extended these findings, suggesting that hTERT played a critical role in HNSCC malignant advances. Importantly, in our study, we found that upregulation of hTERT positively conferred protective activity in response to dead signal in normal and cancer cells, which indicated an essential role of hTERT for cell survival upon chemotherapeutic stimuli in cancer cells.

Evasion of apoptosis is a hallmark of most human tumors and constitutes an important clinical problem [33]. Multiple chemotherapeutic agents including cisplatin act against cancers through inducing apoptosis; however, these agents only provide limited survival advantages due to the significant chemoresistance of HNSCC cells. In the present study, we found that ectopic expression of hTERT increased chemoresistance to cisplatin-induced death and contributed to lower rates of apoptosis, whereas cellular depletion of hTERT substantially sensitized cancer cells and caused higher rates of apoptosis. Our data also showed that hTERT protects cells from death through triggering the AKT/ERK-p53-bcl-2 cascades to inhibit the expression of proteolytic cleavage of caspase-3 and PARP *in vitro*. More importantly, overexpression of hTERT promoted the growth and tumorigenicity of Cal27 cells, significantly impaired the antitumor effect of cisplatin in mouse model and caused altered expression of downstream signaling molecules. Thus, our findings provided a new insight into a possible mechanism that by which hTERT modulated chemosensitivity of HNSCC cells.

Recent study has shown that wide-type p53 exerts distinct functions to promote apoptosis in the context of damage stimuli and suppress the tumorigenesis [34]. There is an evidence also showed increased expression of wild-type p53 could use distinct pathways to repress telomerase activity through inhibition of c-Myc or E-box/E2F pathways in human and mouse cells, respectively [35]. Despite the mutant p53 lost its effect of tumor suppression and obtained oncogenic function in cancer cells, it still retained the ability to drive apoptosis when receiving death stimuli. However, in the present study, our data showed that ectopic hTERT significantly reduced the level of p53 to protect cells from apoptosis, while silencing hTERT represented a reverse trend, identifying p53 as a mediator to regulate the expression of bcl-2, suggesting a close bidirectional regulation exists between hTERT and p53. Thus, targeting TERT could be as a potential strategy to negatively regulate the expression of p53 and induce apoptosis in cancer cells.

**Figure 6:**
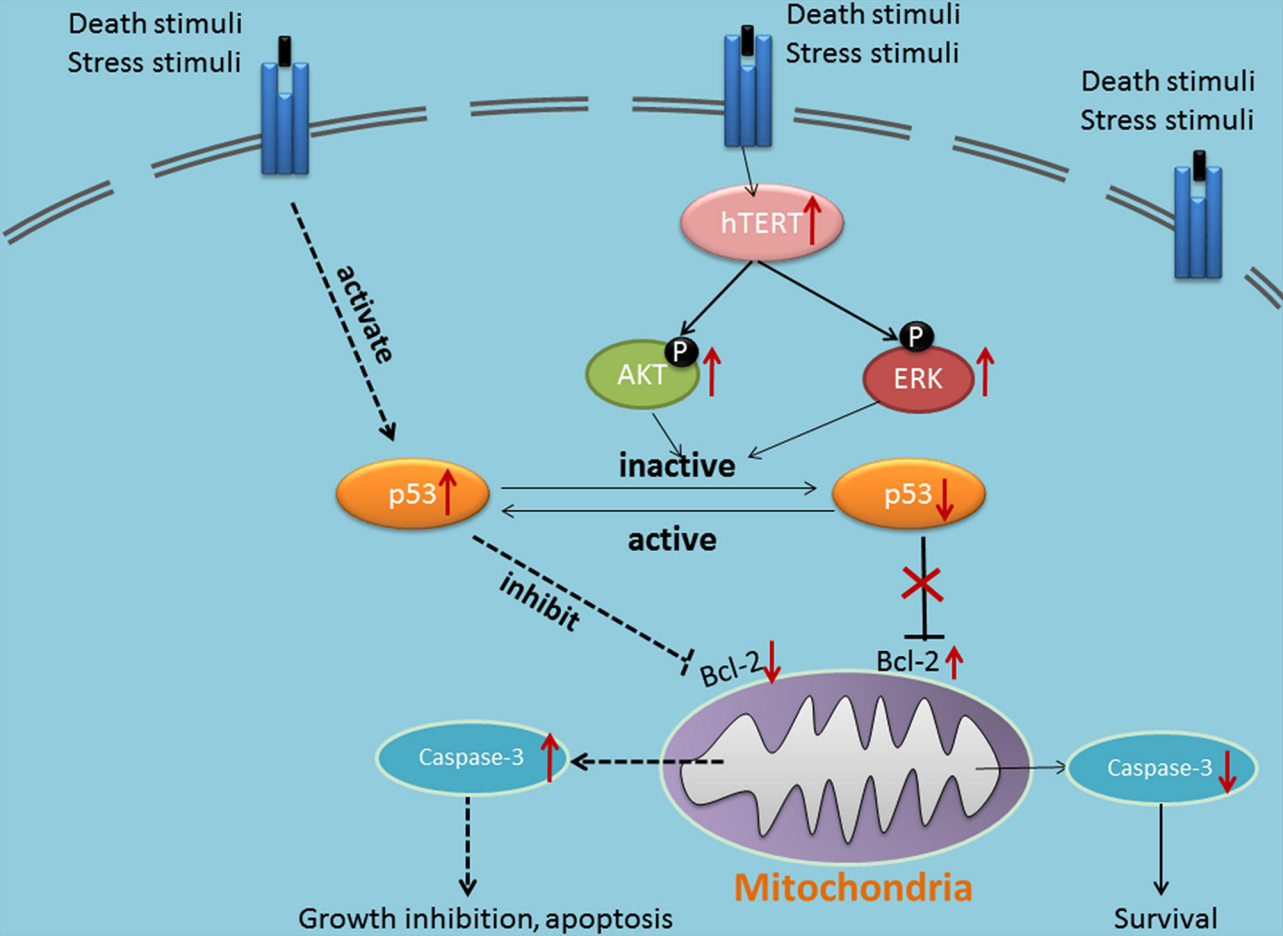
Schematic representation of the anti-apoptosis molecular mechanism of hTERT in regulation the sensitivity of cancer cells to chemotherapy

It is reported that multiple genetic and epigenetic alterations converge on the persistent activation of JAK/STAT3 [36], PI3K/AKT [37], MEK/ ERK [38], Wnt [39]signaling in most cancer lesions. In our current study, our results showed activated phosphorylation of AKT and ERK in hTERT-overexpressing cells, as well as AKT and ERK inactivation in hTERT-depleted cancer cells. Furthermore, we detected that endogenous p-AKT and p-ERK rather than p53 could physically interact with endogenous hTERT in hTERT precipitates by co-IP assays in Cal27 cells. Conclusively, these results demonstrate that upregulated hTERT activates AKT/ERK signaling pathways and supports cell survival through modulation of p53 in HNSCC (Figure 6).

In summary, for the first time, this study reveals a mechanism by which blockade of telomerase reverse transcriptase could enhance chemosensitivity through inhibition of AKT/ERK-p53 signaling pathways. These results provide new insights into the mediators of apoptotic response in HNSCC, which will enable the development of novel antitumor strategies. In this context, targeting hTERT to enhance chemosensitivity in combination of conventional chemotherapies might represent new and promising strategies in head and neck cancers.

## MATERIALS AND METHODS

### Clinical samples and clinical staging system

A total of 57 paraffin-embedded HNSCC samples were obtained from the archives of the Department of Pathology of the Hospital of Stomatology, Sun Yat-sen University, Guangzhou, China. Of the 57 HNSCC samples, we divided them into two independent sets of groups for the experiments. Control group contained 32 well- and 5 moderately-differentiated HNSCCs from 37 patients who did not take any preoperative treatments including chemoradiotherapy, while treated group was comprised of 15 well- and 5 moderately-differentiated paraffin-embedded HNSCCs tumor tissues from 20 patients receiving induction chemotherapy before surgery. All of the samples were treated by surgical excision. For the use of these clinical materials for research purposes, prior patient's consent and approval from the Institute Research Ethics Committee was obtained. The disease stages of all the patients were classified according to Union for International Cancer Control (UICC 2010).

### Cell cultures, plasmids, stable transfection and reagents

Two HNSCC cell lines (Cal27, SCC25) were purchased from American Type Culture Collection (ATCC, USA). Cal27 cells were cultured in Dulbecco's Modified Eagle Medium (Gibco) supplemented with 10% fetal bovine serum (FBS, Hyclone, USA); SCC25 cells were maintained in 1:1mixture of Dulbecco's Modified Eagle Medium and Ham F12 medium (Gibco) supplemented with 10% FBS and 400 ng/ml hydrocortisone (Sigma, USA). Primary cell culture of human oral epithelial cells (HOE) were established as described previously [23]. Briefly, fresh biopsies were obtained from a healthy human adult undergoing third molar extraction. Following by incubation with 2 μg/ml Dispase solution (Roche, Germany) overnight at 4°C, the epidermis were gently separated from the dermis and cut into small pieces, then trpsinized for 5 minutes at 37°C in 0.125% trypsin/0.01 mM EDTA. After vigorous pipetting, the cell suspension was centrifuged for 5 min at 1000 g and the cell pellet was resuspended in keratinocyte serum-free medium (K-SFM, Gibco).

The pCMV and pCMV/hTERT constructs have been previously described [23]. The hTERT gene was introduced into HOE or Cal27 cells by infecting cells with a lentiviral vector pCMV-hTERT. Control cells were infected with the empty lentiviral vector pCMV. Infected cells were subcultured and subjected to drug selection (0.5 and 1 μg/ml puromycin) for 3–5 days, respectively. For depletion of hTERT, the pSIH-H1-puro-construct (System Bioscience, SBI) containing hTERT short hairpin RNAs(shRNA) was generated by cloning the following hTERT-specific RNAi target sequences into pSIHF-H1: hTERT shRNA: 5-GACGGTGTGCACCAACATCTA-3. Briefly, SCC25 cells silencing hTERT were generated by infection with hTERT shRNA lentiviruses, and selected by treatment with 0.5 μg/ml puromycin for 10 days, beginning from 48 h after infection. cisplatin (Sigma, USA) was dissolved in Dimethylformamide (DMF, Sigma, USA), was used to treat cells at indicated final concentrations and times.

### SA-Δ-galactosidase assay

For the SA-Δ–galactosidase assay, the senescence detection kit (Beyotime, China) was used to stain the different passages of pCMV vector or pCMV/hTERT overexpressing HOE cells and determined the SA-Δ-galactosidase activity. Cells were seeded in a 6-well plate and grown for 48 h, then washed with PBS, fixed in 4% paraformaldehyde for 20 minutes at room temperature. The cells were washed again with PBS twice and immersed overnight in the staining solution at 37°C. Senescent cells were identified as blue-stained cells under an IX71 inverted microscope (Olympus, Japan) according to the manufacturer's instruction.

### Cell survival assay

Cell survival was determined using MTT assay to assess the sensitivity of cells to anticancer drugs. Cells were seeded at a density of 5 × 10^3^ cells per well in 96-well culture plates, incubated overnight to allow for cell attachment, then cisplatin was added at a concentration range of 0–100 μM. Cells were treated at required times (48 h–72 h) and incubated with MTT reagent (Sigma, USA). The absorbance was measured in each well with the Microplate Reader at a wavelength of 570 nm. Each experimental point was determined in triplicate.

### Immunohistochemical analysis

Immunohistochemistry was done to examine hTERT expression in 57 human HNSCC tissue specimens, hTERT was detected using a rabbit polyclonal antibody against hTERT (1:25, Santa Cruz, USA). The IHC procedure was as previously described [23]. Interpretation of IHC was made independently by two specialists and the mean cytoplasmic and nuclear hTERT staining intensity (SI; 0, no staining; 1, weak; 2, moderate; 3, strong), labeling indices (LIs, defined as the percentage of positive cells in total cells), and mean labeling scores (LSs, defined as LI × SI) in HNSCC samples with or without chemotherapy were calculated and compared between them.

### Cell cycle and apoptosis analysis

Cell-cycle status was analyzed using DNA content quantitation assay (KeyGEN, China). The cells were washed with PBS thrice, fixed in 70% ethanol at 4°C overnight and stained for total DNA content with RNase A and propidium iodide staining buffer (BD, San Diego, CA, USA) according to the manufacturer's instructions. A minimum of 10000 cells were acquired per sample and cell cycle distribution was analyzed using a flow cytometer (Becton Dickinson, San Jose, CA, USA) and ModFit software V3.0 (Verity Software House, Topsham, ME, USA). The experiments were repeated for three times.

For apoptosis analysis, quantification of apoptotic cells was performed with Annexin-V-APC/7-AADApoptosis Detection Kit (KeyGEN, China). HNSCC cells were treated with cisplatin (6.25 μM) for required times (48 h–72 h). The cells were collected and stained according to the manufacturer's instructions. The apoptosis data acquisition and analysis was performed by a FACSCalibur flow cytometer. Basal apoptosis were identically determined on control cells. All of the samples assayed were in triplicates.

### Intracellular mitochondria-labeled detection

Live pCMV vector or pCMV/hTERT overexpressing HOE cells were labeled with probes for mitochondria using MitoTracker^®^ Red CM-H_2_XRos (Invitrogen), which is a red-fluorescent dye (Ex/Em ~579/599 nm) that stains mitochondria in live cells and its accumulation is dependent upon membrane potential. Cells were seeded onto the glass slides for 24 h, then added prewarmed staining solution and incubated at 37°C for 45 minutes, washed with PBS, fixed in 4% paraformaldehyde for 20 minutes and permeabilized with 0.3% TritonX-100, nuclear was stained with DAPI. Immunofluorescence was detected by confocal laser microscope (Olympus, Japan).

### Western blot analysis

Total cellular proteins were extracted with RIPA lysis buffer. BCA method was used for protein quantification. The aliquots were separated on SDS-PAGE and transferred to a PVDF membrane. The following primary antibodies were used: rabbit anti-hTERT (1:1000, Abcam, MA, USA); mouse anti-p53, rabbit anti-AKT, rabbit anti-p-AKT(Ser473), rabbit anti-ERK1/2, rabbit anti-p-ERK1/2, rabbit anti-PARP, rabbit anti-bcl-2 (1:1000, Cell Signaling Technology, USA); mouse anti-capase-3 (1:1000, Santa Cruz, USA); anti-GAPDH (1:2000, KangChen Bio-tech, MA, China). The protein-antibody complexes were detected by horseradish-conjugated secondary antibodies followed by the enhanced chemiluminescence (ECL) western blotting detection reagents (Pierce, Rockford, USA).

### Co-Immunoprecipitation assay

Total cell lysates were prepared in ice-cold IP lysis buffer (Beyotime, China), and the supernatant of cell lysate corresponding to 1 mg of total protein was precleared by Protein G Plus/Protein A-agarose beads (Millipore, USA) to minimize nonspecific binding. Then the precleared supernatant was divided into three parts, two of which were incubated with 2 μg of anti-hTERT antibody or anti-IgG antibody at 4°C for 1 h separately, followed by incubation with protein G Plus/Protein A- agarose beads overnight at 4°C under gentle agitation. The bound proteins were washed thrice with lysis buffer and dissociated with the beads via boiling and centrifugation. The collected proteins were suspended in 60 μl 1 × SDS loading buffer. The expression of p-AKT, p-ERK and p53 proteins was then analyzed by standard immunoblotting.

### Mice xenograft studies

Six-week-old female BALB/c nude mice were purchased from the Center of Experimental Animal of Guangzhou University of Chinese Medicine (Guangzhou, China). All experimental procedures were approved by the Institutional Animal Care and Use Committee of Sun Yat-sen University. To establish tumor xenografts, the BALB/c nude mice were subcutaneously injection of Cal27/Vector or Cal27/hTERT cells (1 × 10^7^) into the right flank. Xenograft tumors were examined once every three days. After 10 days, two different kinds of tumor bearing mice were randomized respectively into two groups (six mice per group), and treated by intraperitoneal injection (ip) with cisplatin (2 mg/kg) or control diluent every day. Tumor size was measured using a slide caliper and tumor volume was determined by the formula (L × W^2^)/2 (L indicates the length-diameter and W the width-diameter of the tumor). 10 days after injection, animals were killed at the indicated time points, and tumors were isolated for histologic and immunohistochemical evaluation, as described previously. The following primary antibodies were used for IHC: rabbit anti-hTERT (1:200, Abcam, MA, USA), mouse anti-p53 and mouse anti-bcl-2 (ZSGB-BIO, China), rabbit anti-cleaved caspase-3 (1:2000, Cell Signaling Technology, USA).

### Statistical analysis

Data are represented as Mean ± SD. The data were analyzed by the Student's *t* test using the SPSS 15.0 software to determine their significant differences. *P < 0.05 was considered statistically significant.

## SUPPLEMENTARY FIGURE


