# Supplementary material for: Blockade of telomerase reverse transcriptase enhances chemosensitivity in head and neck cancers through inhibition of AKT/ ERK signaling pathways
Source: Oncotarget. 2015 Oct 16;6(34):35908–21. doi: 10.18632/oncotarget.5468 (PMC4742150; doi:10.18632/oncotarget.5468)
Supplement: Supplementary file 1 [file oncotarget-06-35908-s001.pdf]

SUPPLEMENTARY FIGURE

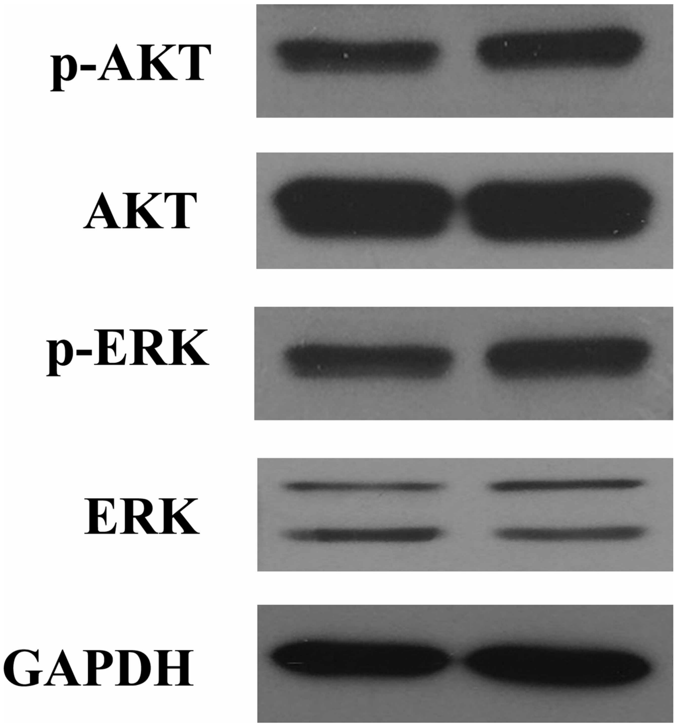

Supplementary Figure S1: Ectopic expression of hTERT activates the AKT/ERK pathway in HOE cells. The total cell lysates were analyzed for phosphorylated AKT, total AKT, phosphorylated ERK and total ERK levels by Western blot analysis. Anti-GAPDH was used as loading control.
